# Transfer of Maternal Antibodies against Avian Influenza Virus in Mallards (*Anas platyrhynchos*)

**DOI:** 10.1371/journal.pone.0112595

**Published:** 2014-11-11

**Authors:** Jacintha G. B. van Dijk, A. Christa Mateman, Marcel Klaassen

**Affiliations:** 1 Department of Animal Ecology, Netherlands Institute of Ecology (NIOO-KNAW), Wageningen, The Netherlands; 2 Centre for Integrative Ecology, School of Life and Environmental Sciences, Deakin University, Geelong, Australia; University of Milan, Italy

## Abstract

Maternal antibodies protect chicks from infection with pathogens early in life and may impact pathogen dynamics due to the alteration of the proportion of susceptible individuals in a population. We investigated the transfer of maternal antibodies against avian influenza virus (AIV) in a key AIV host species, the mallard (*Anas platyrhynchos*). Combining observations in both the field and in mallards kept in captivity, we connected maternal AIV antibody concentrations in eggs to (i) female body condition, (ii) female AIV antibody concentration, (iii) egg laying order, (iv) egg size and (v) embryo sex. We applied maternity analysis to the eggs collected in the field to account for intraspecific nest parasitism, which is reportedly high in *Anseriformes*, detecting parasitic eggs in one out of eight clutches. AIV antibody prevalence in free-living and captive females was respectively 48% and 56%, with 43% and 24% of the eggs receiving these antibodies maternally. In both field and captive study, maternal AIV antibody concentrations in egg yolk correlated positively with circulating AIV antibody concentrations in females. In the captive study, yolk AIV antibody concentrations correlated positively with egg laying order. Female body mass and egg size from the field and captive study, and embryos sex from the field study were not associated with maternal AIV antibody concentrations in eggs. Our study indicates that maternal AIV antibody transfer may potentially play an important role in shaping AIV infection dynamics in mallards.

## Introduction

Maternal antibodies provide protection against pathogens in the early stages of a chick's life [1]. These antibodies are relatively short-lived (from a few days to more than a month), with the duration of their presence being dependent on species [2], [3], after which a chick's own antibody production needs to take over [4], [5]. Maternal antibodies can be of crucial importance for a chick's initial humoral defence and growth rate, potentially increasing its survival during the early stages of its life (for review see [6]). Moreover, these maternal antibodies can have considerable impact on pathogen dynamics at the population level, reducing the proportion of susceptible individuals in a host population [1]. Hence, studying maternal antibodies in any host-pathogen system is important to evaluate their potential role in shaping pathogen dynamics in animal populations.

Low-pathogenic avian influenza virus (AIV), a pathogen that circulates naturally in wild birds, is highly studied, though the role of maternal antibodies in AIV infection dynamics in wild birds has been largely neglected. The limited studies on maternal antibodies against AIV in wild birds are conducted in gulls [7], a family of birds that acts as a reservoir host for AIV subtypes H13 and H16 [8], [9]. Circulating antibodies to H13 AIV in adult ring-billed gulls (*Larus delawarensis*) ranged between 82 and 92%, with antibody prevalence in 3-wk-old chicks ranging between 5 and 30% [10]. In yellow-legged gulls (*Larus michahellis*), 81% of females had circulating AIV antibodies and 51% of their eggs received these antibodies maternally [11]. In this species it was shown that maternal AIV antibodies in eggs reflected the circulating AIV antibodies of the laying females, and that the first-laid eggs received highest levels of maternal antibodies [12]. These findings in gulls indicate a considerable transfer of maternal AIV antibodies to eggs, raising the possibility that maternal antibody transfer might also play a vital role in AIV infection dynamics in other host species.

Many avian studies showed a strong correlation between maternal antibodies in egg yolk and/or chicks and circulating antibody levels in mothers [5], [13]–[16]. For instance, for *Borrelia* (Lyme disease agent) antibody concentrations in kittiwake (*Rissa tridactyla*) eggs and female blood sera correlated positively [17]. Female body condition may positively impact maternal antibody transfer [15], [18], which may, at least in part, be due to the positive effect of body condition on female antibody levels [18]. Additional correlations between maternal antibody levels in eggs have been shown with female age and colour [19],[20], breeding density and sexual attractiveness of male mates [21], [22], egg colour [23], [24], laying order [25], [26] and the sex of offspring [16,^27^,28].

The aim of our study was to investigate maternal antibody transfer in a key AIV host species, the mallard (*Anas platyrhynchos*) [29]. In this correlative study we examined how several covariates correlated with the concentration of maternal AIV antibodies in eggs. We sampled free-living female mallards together with their eggs, connecting yolk maternal AIV antibody concentrations to (i) female body condition, (ii) female AIV antibody concentration, (iii) egg size and (iv) embryo sex. Additionally, we examined the correlation between yolk maternal AIV antibody concentrations and (v) egg laying order in a study with captive females and their eggs. In the light of previous findings in other avian species, we hypothesized that high concentrations of maternal AIV antibodies in eggs would correlate with high AIV antibody levels in females.

## Materials and Methods

### Ethics statement

The field study was conducted in a woodland area in the Alblasserwaard (51°52′38″N, 4°43′26″E), the Netherlands. Free-living mallards were caught under the Flora and Fauna permit (FF/75A/2010/011), issued by the Dutch Ministry of Economic Affairs, since the mallard is a protected species in the Netherlands. Handling and sampling of free-living mallards was approved by the Animal Experiment Committee (protocol CL10.02) of the Royal Netherlands Academy of Arts and Sciences (KNAW). The captive study was conducted in the outdoor aviary at the Netherlands Institute of Ecology (NIOO-KNAW) in Heteren (51°57′26″N, 5°44′33″E), the Netherlands, who granted permission to perform this study. Approval from the KNAW Animal Experiment Committee (addendum protocol CL10.02) was given to handle and sample mallards in captivity. Free-living mallards were released into the wild after sampling, whereas captive mallards were set on an *ad libitum* feeding regime in the outdoor aviary after the study. All efforts were made to minimize animal suffering throughout the studies.

### Field study

From April until June 2010, 67 free-living female mallards were caught from their nest with a sweep net. Females were caught during incubation to lower the chance of nest abandonment. To reduce the risk of including nests with eggs that had primarily been dumped there by other females, we only sampled females with a clutch of 13 eggs or less [30]. Captured females were marked with a metal ring and categorized as juvenile (±1 year; first reproduction) or adult (>1 year) based on plumage characteristics [31]. To index body size, we measured tarsus length (nearest 0.01 mm [32]), head+bill length (nearest 0.1 mm) and wing length (maximum wing chord, nearest 1 mm [33]). A digital balance was used to measure body mass (nearest 1 g). Blood samples (<1 ml, 2% of the circulating blood volume) were collected from the brachial vein for detection of antibodies to AIV. Blood was allowed to clot for approximately 6 h before centrifugation to separate serum from red blood cells [34]. Ethanol (70%) was added to the red blood cells, and together with the sera samples, stored at −20°C until analysis.

Per clutch, two randomly chosen eggs were collected to assess maternal AIV antibody concentration in egg yolk. Of each egg, the length (L; nearest 0.01 mm) and breadth (two measurements as eggs are frequently not circular, B_1_ and B_2_; nearest 0.01 mm) were taken to assess egg size. Egg yolks were separated on the day of collection. The size of each embryo was measured with a ruler (nearest 0.01 mm) to account for potential age differences affecting yolk AIV antibody concentration [35]. Egg yolk and embryos were frozen at −20°C until analysis.

### Captive study

In the same period as the field study, we conducted a study with 16 adult female and 10 adult male mallards kept in captivity in an outdoor aviary. All birds were captive bred and either originated from a waterfowl breeder (n = 16; P. Kooy & Sons, 't Zand, the Netherlands) or were bred at the NIOO-KNAW (n = 10). All birds had been kept in the outdoor aviary for at least a year prior to the study. The females were individually marked with colour rings to allow visual recognition.

The outdoor aviary was divided in five compartments: one large compartment (15×13 m) and four smaller compartments (6×13 m). In the large compartment, six females and three males were housed. The smaller compartments contained: two females and two males, three females and one male, three females and two males. Males were assigned to females according to pairs that had already formed before the start of the study. Each compartment was connected to a pond (34×1.5 m), with continuous flowing water for bathing and drinking. The outdoor aviary was surrounded by anti-bird nets and vermin proof mesh wire to prevent (egg) predation. To lower the chance that eggs were laid in a foreign nest, a surplus of nest boxes were provided in each compartment. Birds had access to shelter in the form of tall vegetation surrounding the aviary. Food was provided *ad libitum* and consisted of a mixture of commercial food pellets and seed-based mixed grains.

During egg laying, blood samples were collected from the brachial vein of females to measure concentrations of AIV antibodies. Analogous to the field study, serum was separated from red blood cells, and stored at −20°C until analysis. Female body mass, tarsus and head+bill lengths were measured (wing length was not scored as primary feathers were clipped to prevent flying). Once females started laying eggs, freshly laid eggs were numbered with a nontoxic pen referring to the position within the laying order. Per clutch, we collected four eggs (one fresh egg and three eggs during incubation) to assess a potential change in yolk AIV antibody concentration during the course of incubation. At the day of collection, egg length (L) and two breadth measurements (B_1_ and B_2_) were taken, egg yolks separated, embryos measured and samples frozen at −20°C until analysis.

### Antibody detection

The protocol of Mohammed et al. [36] was followed to prepare egg yolk samples. Once thawed, 0.93 g of egg yolk was diluted 1∶1 in phosphate-buffered saline and homogenized using a vortex shaker. Of the diluted egg yolk suspension, 0.9 ml was placed in a 2 ml tube and an equal volume of chloroform was added and vortexed 20–30 sec. The mixture was incubated at room temperature for 30 min, centrifuged at 4°C 17,949× g (5804R; Eppendorf, Nijmegen, the Netherlands) for 10 min, and the clear supernatant was used in the immunoassay. Of four eggs collected in the field, we were unable to collect sufficient yolk as the embryos were too large and had absorbed most of the yolk.

The presence of antibodies to the highly conserved nucleoprotein of AIV in female serum and egg yolk was tested using a commercially available blocking enzyme-linked immunosorbent assay (bELISA MultiS-Screen Avian Influenza Virus Antibody Test Kit; IDEXX Laboratories, Hoofddorp, the Netherlands) following manufacturer's instructions. Samples were tested in duplicate, with each plate containing two positive and two negative controls. The absorbance (i.e. OD-value) was measured at 620 nm using an infinite M200 plate reader (Tecan Group Ltd, Männedorf, Switzerland). Female serum and egg yolk samples were considered AIV antibody positive if the signal to-noise ratio (i.e. mean OD-value of the sample divided by the mean OD-value of the negative control) was <0.5.

To validate the use of OD-values as a quantitative estimate of antibody concentration, we applied a serial dilution of 10 (randomly selected) AIV antibody positive egg yolks and female sera from the field study. On two bELISA plates, the AIV antibody positive yolks and sera were diluted 1/3, 1/10, 1/30 and 1/100, together with positive and negative controls for each dilution except 1/3. Samples were tested in duplicate and the OD-value measured. Dilution and OD-value were log10-transformed before tested in a linear mixed model (LMM), with individual sample as random factor. There was a strong correlation between the dilution and the OD-value of egg yolk (χ^2^ = 86.791, p<0.001; linear model: y = −0.55x+1.07, r^2^ = 0.73) and female sera (χ^2^ = 68.218, p<0.001; linear model: y = −0.47x+1.23, r^2^ = 0.78; Figure S1). This linear relation suggests that OD-values of yolk and sera can indeed be used as a relative measure of AIV antibody concentration (hereafter called AIV antibody concentration).

Repeatability of OD-values in egg yolk and female sera collected in the field and captive study was 0.49 and 0.70, respectively [37]. Intra-plate repeatability of the ELISA was assessed by calculating the coefficient of variation (CV%) of the OD-values of 50 replicates of positive and negative control samples, which was respectively 10.4% and 3.8%. Inter-plate reproducibility of the ELISA was evaluated using the CV of the OD-values of positive and negative controls on 62 different plates, which was respectively 10.9% and 4.3%. CVs <20% for raw OD-values indicate adequate repeatability of the assay [38].

### Maternity analysis

Given the provisioning of sufficient nest boxes and space, continued monitoring and the observation that no clutches exceeded 13 eggs, we were confident that the females that laid and incubated the eggs in the captive study were the biological females. In the wild, intraspecific nest parasitism, whereby females lay eggs in nests of other conspecifics, is very common among *Anseriformes* [39]. In free-living mallards, 24% of clutches may contain parasitic eggs [40]. Therefore, maternity was assessed for all eggs collected in the field, minus the eggs that did not contain an embryo (n = 3) or had insufficient yolk (n = 4), i.e. 127 eggs in 67 clutches.

DNA was extracted from female red blood cells and embryonic tissue using a Gentra Puregene Kit (Qiagen, Venlo, the Netherlands). A small amount of blood or tissue was transferred into a 1.5 ml tube containing 1000 µl of Cell Lysis Solution and 10 µl Puregene Proteinase K solution. After overnight incubation at 55°C, the remainder of the extraction protocol was completed according to the manufacturer's instructions. At the final step DNA was dissolved in 100 µl DNA Hydration Solution (Qiagen, Venlo, the Netherlands).

Maternity was assigned using eight polymorphic microsatellite markers for mallards (APL 2, APL 11, APL 12, APL 14, APL 23, APL 26, APL 36 [41], APH 17 [42]). PCR was performed in a 10 µl reaction mixture containing 5 µl Multiplex PCR mix (Qiagen, Venlo, the Netherlands), 40 ng DNA and four labelled primers. After amplification PCR products were analysed using an ABI 3130 automated capillary sequencer with a molecular size standard (GeneScan-500 LIZ; Life Technologies, Bleiswijk, the Netherlands). Sizes of the amplification products were determined using commercial software (GeneMapper 4.0; Life Technologies, Bleiswijk, the Netherlands). Using CERVUS 3.0, the combined non-exclusionary probability of the first parent of the given set of markers was 0.99948, and of the second parent 0.99999. Matching females with offspring was based on maximum likelihood, as implemented in CERVUS 3.0 [43]. To estimate the 95% confidence interval for the differences in log-likelihood scores between the genetic and second-most likely mothers, based on known maternal genotype, a simulation (10,000 cycles) was performed using the known distribution of allele frequencies. The proportion of candidate mother samples was 98%. Maternity was assigned when the most likely mother matched the young at least at 7 loci.

### Embryo sexing

The sex of embryos was determined for eggs collected in the field. Embryo gender was identified using the W chromosome-linked CHD-1 gene. Primers P2 and P8 were used following Griffiths et al. [44] and PCR amplifications was carried out in a total volume of 10 µl, containing 5 µl PCR-Mix (Promega, Leiden, the Netherlands), with an extra 0.2 µl MgCl_2_ (25 mM) and 40 ng genomic DNA. PCR was performed on a PTC200 (Biorad, Veenendaal, the Netherlands). PCR product was digested with 1 µl EcoRI restriction endonuclease (20 U/µl) in a total volume of 10 µl [45]. The mixture was incubated at 37°C for 1.5 h, and 2 µl of loading buffer was added to each sample. PCR products were separated by electrophoresis for 120 min at 40 mA in a 2% agarose gel and stained with ethidium bromide.

### Statistical analysis

The field dataset contained 64 biological females (of three clutches the female caught on the nest was not the biological mother) and their (non-parasitic) eggs (n = 115). For 13 clutches only one egg was included in the analysis, as the other eggs contained no embryo (n = 3), had insufficient yolk (n = 4) or the female caught on the nest was not the biological mother (n = 6). The dataset of the captive study contained eight egg laying females, where one female produced two clutches. A total of 33 eggs were collected. For three clutches, one egg during incubation was missing. As an index of body size, we used the first principal component (PC1) of a PC analysis of the biometric measurements. For the field study, PC1 explained 60% of the variance across tarsus, head+bill and wing lengths. Only tarsus and head+bill lengths were used in the PC analysis of the captive study, PC1 explaining 79% of the variance. To assess egg size, the volume (mm^3^) was calculated following Hoyt [46]: 0.000515×L×B_1_×B_2_ (L: length, B_1_ and B_2_: breadth).

All covariates were log10-transformed to meet the assumption of normality. AIV antibody concentrations in egg yolk and female serum were minuslog10-transformed so that high values indicated high concentrations of AIV antibodies. Collinearity between the various covariates for the field and captive study were tested using Pearson correlation (r). In the field study, body mass and female AIV antibody concentration (r = 0.38, p<0.001, r^2^ = 0.14), and body mass and egg volume were correlated (r = 0.43, p<0.001, r^2^ = 0.16; Table S1). All three covariates were retained in the model to test the variance in yolk AIV antibody concentration, because of their relatively weak correlation and to allow comparison with the captive study results (where no collinearity existed between these three factors). There was no correlation between egg laying order and embryo size in the captive study (r = −0.21, t = −1.207, p = 0.237, r^2^ = 0.01).

For both the field and the captive study, we used a LMM to test the association between yolk AIV antibody concentration and female body mass, female AIV antibody concentration, egg volume and all two-way interactions, with clutch as random factor. Additionally, embryo sex was included as an explanatory variable for the field study, whereas egg laying order was included in the model for the captive study. Female size and embryo size were included in the models to adjust respectively body mass for structural size and to account for age differences potentially affecting yolk AIV antibody concentration. Female age was excluded from the analysis of the field study, since most of the females were adults (adult: 86%, juvenile: 3%, unknown: 11%). Full factorial models were tested and model selection was used to assess the better models on the basis of Akaike Information Criterion (AIC) corrected for small sample sizes (AICc [47]). The better models were defined as those models with the least number of parameter within a ΔAIC_c_<2 relative to the best-supported model (i.e. the model with the lowest AIC_c_) (for model selection see Table S2). The significance level (α) equalled 0.05.

All analyses were conducted using R 2.14.1 [48], where package lme4 was used to fit the LMMs [49].

## Results

### Field study

We identified 12 parasitic eggs out of 127 (9%) in nine out of 67 clutches (13%), with both eggs parasitic in three clutches. Prevalence of AIV antibodies in breeding females was 48% (31 out of 64), with 43% (50 out of 115) of the eggs receiving maternal AIV antibodies.

**Figure 1:**
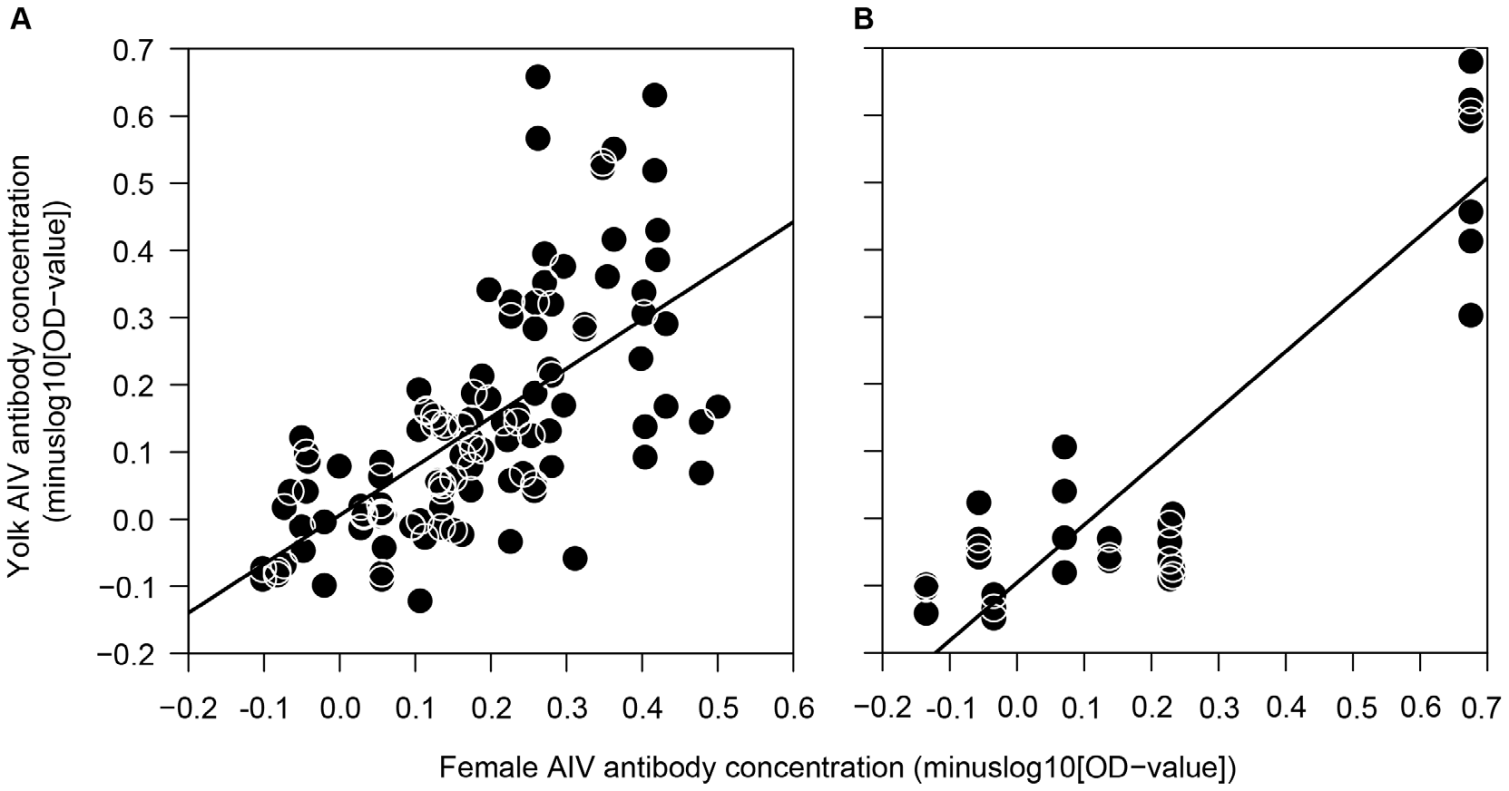
Association between the AIV antibody concentration in egg yolk and female serum from (A) the field study and (B) the captive study. Note: axes are minuslog10-scaled.

Of all investigated factors, only female AIV antibody concentration was correlated with AIV antibody concentration in egg yolk (Table 1): high antibody concentrations in females corresponded with high concentrations in eggs (Figure 1A). Female body mass, corrected for size, was not associated with yolk AIV antibody concentration (Table 1). Also egg volume and embryo sex were not correlated with concentrations of AIV antibodies in egg yolk, nor was there an effect of embryo size on yolk AIV antibody concentration (Table 1).

**Table 1:** Model output of the best-supported model used to analyse the variation in concentrations of maternal antibodies against AIV in egg yolk in the field and captive study.

	Field study^1^		Captive study^2^	
Covariate	?^2^	p-value	?^2^	p-value
Body mass	0.005	0.943	1.234	0.267
Female size	0.181	0.670	0.559	0.439
AIV OD-value female serum^3^	30.434	**<0.001**	16.647	**<0.001**
Egg volume	0.072	0.789	1.218	0.270
Embryo sex	1.346	0.246		
Embryo size	1.148	0.700	2.079	0.149
Egg laying order			8.145	**0.005**

1 Free-living mallards and eggs.

2 Captive mallards and eggs.

3 Relative concentration of antibodies against avian influenza virus (AIV) in female sera.

### Captive study

**Figure 2:**
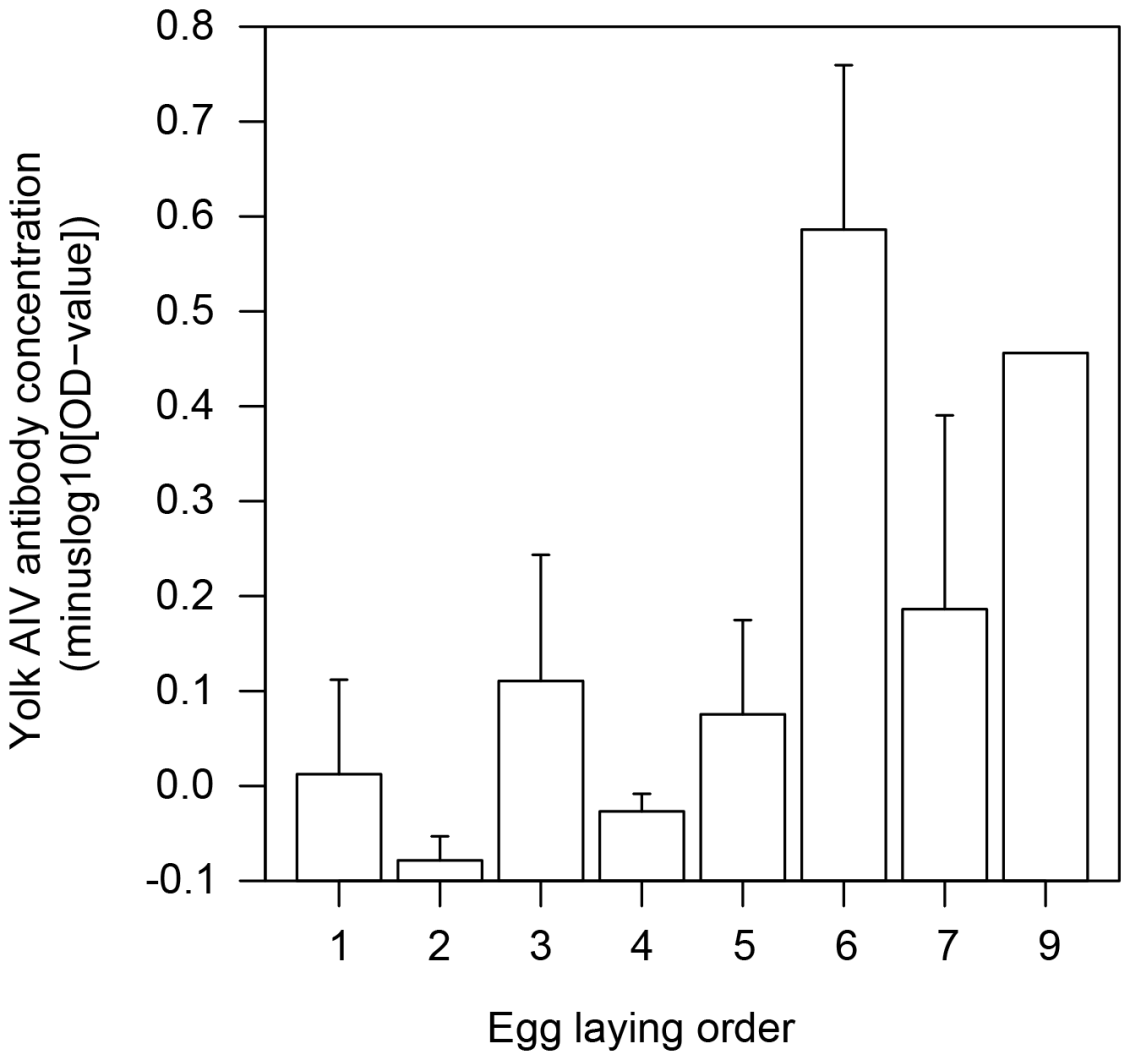
AIV antibody concentration in egg yolk in relation to egg laying order from the captive study. Note: x-axis is minuslog10-scaled.

AIV antibody prevalence in breeding females was 56% (5 out of 9) and in eggs 24% (8 out of 33). Similar to the field study, AIV antibody concentrations in egg yolk were positively correlated with female AIV antibody concentrations (Figure 1B, Table 1). Also egg laying order was correlated with yolk AIV antibody concentration (Table 1): eggs that were laid later in the laying sequence had higher antibody concentrations than first laid eggs (Figure 2). Female body mass was not associated with AIV antibody concentration in egg yolk, with no effect of female size (Table 1). There was also no correlation between egg volume and yolk AIV antibody concentration, nor an effect of embryo size (Table 1).

## Discussion

In free-living mallards, nearly half of the eggs received maternal AIV antibodies. Although maternal AIV antibodies were not measured in chicks, several studies showed positive correlations between maternal antibodies in females, eggs and chicks [14], [15]. It is thus likely that a considerable proportion of newly hatched mallard chicks in our study site were protected against AIV during their first weeks by means of maternal AIV antibodies. In mallard chicks, maternal immunoglobulin IgY decreases after five days post-hatch, reaching minimum levels at about 14 days of age [50]. If indeed a considerable proportion of mallard chicks received maternal AIV antibodies, this could reduce the number of susceptible individuals in the population, potentially affecting the dynamics of AIV infections in mallards.

In the captive study, only a quarter of the mallard eggs received maternal AIV antibodies, whereas at least half of the females had circulating AIV antibodies. This may be considered surprising, since females in captivity had unlimited access to food and thus resources to support immune function. Several studies showed that food supplementation may increase and food limitation may decrease the levels of maternal antibodies that are transferred into eggs [18], [51]. However, negative effects of supplementary feeding on maternal antibody transfer have also been recorded [52]. A potential reason why not all eggs in our captive study received maternal AIV antibodies could be related to stress: living in captivity may induce stress (i.e. high ratios of heterophils to lymphocytes [53]), which could negatively affect maternal antibody transfer into eggs [15].

As hypothesized, the circulating AIV antibody concentrations in females correlated with the AIV antibody concentrations in egg yolk in both the field and the captive study. Our results are in accordance with earlier findings of maternal AIV antibody transfer in free-living gulls [12]. An experimental study in breeder ducks also showed that AIV antibody titres in egg yolk and serum were highly correlated [54]. Transferring high levels of maternal antibodies to chicks likely increases the chicks' period of protection against pathogen infection [55], with a strong relationship between maternal antibody levels at hatching and the period of persistence of these antibodies [14]. For instance, poultry hatchlings with high initial maternal antibody titres against chicken anaemia agent maintained antibodies one to three weeks longer compared to hatchlings with low initial maternal antibody titres [56]. This protective effect of maternal AIV antibodies may also affect the development of a chick's own immune system by blocking the immune response when chicks are exposed to pathogens [1]. Garnier et al. [3] showed that three week old Cory's shearwater (*Calonectris diomedea*) chicks with maternal antibodies against Newcastle disease virus (NDV) did not increase their antibody levels when vaccinated with NDV, whereas chicks without maternal NDV-antibodies did.

We found a positive correlation between egg laying order and maternal AIV antibody concentration in our captive study. An increase in maternal antibodies over the laying sequence may function as a maternal tool to mitigate the negative consequences for the last-hatched chick(s) [57]. On the other hand, females may also enhance the levels of maternal antibodies in first-laid eggs to increase the competitive disparity among siblings when food availability is insufficient to rear the entire brood [58]. Within-clutch variation in maternal antibodies can be a flexible mechanism by which females can influence sibling competition. This evolutionary interpretation is, however, strongly related to asynchronous hatching [59]. In mallards, hatching of chicks is synchronous [60], which likely reduces the need to deposit more maternal antibodies in last-laid eggs to increase chick survival. On the other hand, mallard chicks from last-laid eggs may have had to invest extra (energy) resources in speeding up embryonic development and complete the hatching process to allow for synchronous hatching [61], potentially lowering their body mass and negatively influencing their survival [62]. To compensate for this fitness loss females may preferentially provide last-laid eggs with (extra) maternal antibodies.

According to evolutionary theory parental investment should be adjusted to the reproductive value of current eggs or offspring [63]. Based on our results, one would thus conclude that last-laid eggs are apparently of greater reproductive value than first-laid eggs, since last-laid eggs received higher concentrations of AIV antibodies than first-laid eggs. Possibly, this is related to a higher risk of predation for first-laid compared to last-laid eggs, since the former are exposed to predation risk longer than the last-laid eggs before incubation starts.

In both field and captive study, female body mass was not correlated with yolk AIV antibody concentrations. Also in female gulls no correlation was found between their body mass and AIV antibody levels in their eggs [12]. Yet, other avian studies did find a positive correlation between the two [26], [64]. We also found no correlation between yolk maternal AIV antibody concentration and egg size in mallards. A similar non-significant correlation between these two is found in other bird studies [15]. Grindstaff et al. [13] showed that female Japanese quail (*Coturnix japonica*) that were fed low protein diets produced smaller eggs, but maternal antibody concentration in egg yolk was not affected. In contrast to our prediction, eggs with female embryos did not receive higher concentrations of maternal AIV antibodies than males. Despite lower survival in females [65], mallards seemingly do not favour a particular offspring sex by allocating higher concentrations of maternal AIV antibodies. A potential explanation could be that with each clutch mallards produce many offspring of both sexes as they lay large clutches (average clutch size 9 to 13 eggs [60]). In contrast to bird species that produce clutches half the size of that of mallards, and thus produce fewer offspring of each sex per clutch, making it profitable to allocate higher concentrations of maternal antibodies to a particular offspring sex in favour [16], [27]. In both field and captive study, embryo size had no effect on the AIV antibody concentration in egg yolk. This indicates that the concentration of AIV antibodies in egg yolk does not change during incubation, which is similar as is found with IgY levels in chicken eggs [35].

In conclusion, by investigating transfer of maternal AIV antibodies in mallards as a key AIV host species, we demonstrated that in free-living mallards nearly half of the eggs received maternal AIV antibodies. Concentrations of maternal AIV antibodies in mallard eggs are positively correlated with circulating AIV antibody concentrations in females and laying order. With this study, we highlight the importance of studying maternal AIV antibody transfer in wild birds, which may play an important role in shaping AIV infection dynamics in host populations.

## Supporting Information

Figure S1 **OD-values (i.e. ELISA absorbance values) as a function of dilution factor.** (A) Egg yolk and (B) female serum. Lines represent significant least square regression lines.
(TIF)

Table S1 **Correlation coefficients between the (continuous) covariates of interest for the field and captive study.** Covariates that are significantly correlated are depicted in bold.
(PDF)

Table S2 **Model selection to assess the better models to test the relationship between maternal avian influenza virus (AIV) antibody concentration in egg yolk and the covariates of interest for the field and captive study.** The better models are shown in bold.
(PDF)
