# Supplementary figures and images for: Transfer of Maternal Antibodies against Avian Influenza Virus in Mallards (Anas platyrhynchos)
Source: PLoS One. 2014 Nov 11;9(11):e112595. doi: 10.1371/journal.pone.0112595 (PMC4227685; doi:10.1371/journal.pone.0112595)

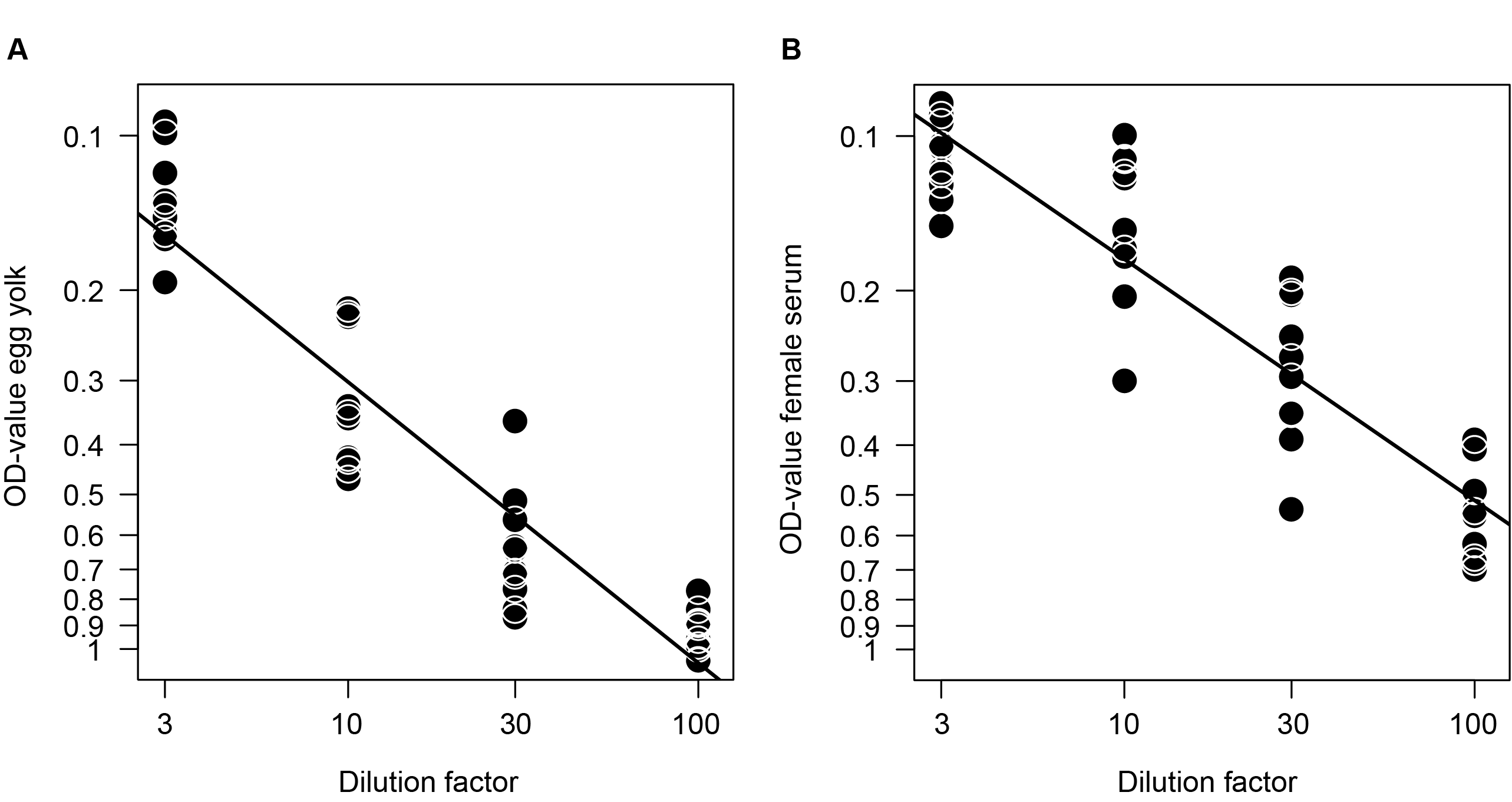

Supplement: Figure S1 — OD-values (i.e. ELISA absorbance values) as a function of dilution factor. (A) Egg yolk and (B) female serum. Lines represent significant least square regression lines. (TIF) [file pone.0112595.s001.tif]
