# Supplementary material for: Transfer of Maternal Antibodies against Avian Influenza Virus in Mallards (Anas platyrhynchos)
Source: PLoS One. 2014 Nov 11;9(11):e112595. doi: 10.1371/journal.pone.0112595 (PMC4227685; doi:10.1371/journal.pone.0112595)
Supplement: Table S1 — Correlation coefficients between the (continuous) covariates of interest for the field and captive study. Covariates that are significantly correlated are depicted in bold. (PDF) [file pone.0112595.s002.pdf]

Table S1. Correlation coefficients between the (continuous) covariates of interest for the field and captive study. Covariates that are significantly correlated are depicted in bold.

	Field study[1]				Captive study[2]			
Covariate	r	t-value	p-value	r^2	r	t-value	p-value	r^2
Body mass * AIV OD-value female serum[3]	0.38	4.378	**<0.001**	0.14	0.14	0.794	0.433	0.05
Body mass * Egg volume	0.43	5.115	**<0.001**	0.16	0.26	1.523	0.138	0.04
Body mass * Egg laying order					0.13	0.689	0.496	-0.02
AIV OD-value female serum * Egg volume	0.07	0.752	0.455	0.00	-0.28	-1.651	0.109	0.05
AIV OD-value female serum * Egg laying order					0.28	1.647	0.110	0.05
Egg volume * Egg laying order					-0.31	-1.797	0.082	0.07

[1]Free-living mallards and eggs

[2]Captive mallards and eggs

[3]Relative concentration of antibodies against avian influenza virus (AIV) in female sera
