# Supplementary material for: Transfer of Maternal Antibodies against Avian Influenza Virus in Mallards (Anas platyrhynchos)
Source: PLoS One. 2014 Nov 11;9(11):e112595. doi: 10.1371/journal.pone.0112595 (PMC4227685; doi:10.1371/journal.pone.0112595)
Supplement: Table S2 — Model selection to assess the better models to test the relationship between maternal avian influenza virus (AIV) antibody concentration in egg yolk and the covariates of interest for the field and captive study. The better models are shown in bold. (PDF) [file pone.0112595.s003.pdf]

Table S2. Model selection to assess the better models to test the relationship between maternal avian influenza virus (AIV) antibody concentration in egg yolk and the covariates of interest for the field and captive study. The better models are shown in bold.

Study	Model	Covariate	df	logLik	AIC_c	ΔAIC_c
Field study[1]	6	m[3] + s[4] + a[5] + v[6] + es[7] + esi[8] + m*a	10	106.649	-193.3	0.00
	5	m + s + a + v + es + esi + m*a + m*v	11	107.516	-193.0	0.27
	4	m + s + a + v + es + esi + m*a + m*v + m*es	12	107.998	-192.0	1.30
	**7**	**m + s + a + v + es + esi**	**9**	**104.906**	**-191.8**	**1.49**
	3	m + s + a + v + es + esi + m*a + m*v + m*es + a*v	13	108.187	-190.4	2.92
	1	m + s + a + v + es + esi + m*a + m*v + m*es + a*v + a*es + v*es	15	109.483	-189.0	4.33
	2	m + s + a + v + es + esi + m*a + m*v + m*es + a*v + a*es	14	108.390	-188.8	4.52
Captive study[2]	**7**	**m + s + a + v + l[9] + esi**	**9**	**39.438**	**-60.9**	**0.00**
	6	m + s + a + v + l + esi + m*a	10	39.438	-58.9	2.00
	5	m + s + a + v + l + esi + m*a + m*v	11	40.067	-58.1	2.74
	4	m + s + a + v + l + esi + m*a + m*v + m*l	12	40.101	-56.2	4.67
	3	m + s + a + v + l + esi + m*a + m*v + m*l + a*v	13	40.122	-54.2	6.63
	2	m + s + a + v + l + esi + m*a + m*v + m*l + a*v + a*l	14	40.927	-53.9	7.02
	1	m + s + a + v + l + esi + m*a + m*v + m*l + a*v + a*l + v*l	15	40.933	-51.9	9.01

[1]Free-living mallards and eggs

[2]Captive mallards and eggs

[3]m: body mass

[4]s: female size (first principal component [PC1] of tarsus, head+bill and wing lengths [no wing in captive study])

[5]a: relative concentration of antibodies against avian influenza virus in female sera

[6]v: egg volume (0.000515 x L x B_1 x B_2 [L: length, B_1, B_2: breadth])

[7]es: embryo sex

[8]esi: embryo size

[9]l: laying order
